# In Vivo Determination of Fluctuating Forces during Endosome Trafficking Using a Combination of Active and Passive Microrheology

**DOI:** 10.1371/journal.pone.0010046

**Published:** 2010-04-06

**Authors:** Damien Robert, Thi-Hanh Nguyen, François Gallet, Claire Wilhelm

**Affiliations:** Laboratoire Matière et Systèmes Complexes (MSC), UMR 7057 CNRS et Université Paris Diderot, Paris, France; The University of Manchester, United Kingdom

## Abstract

**Background:**

Regulation of intracellular trafficking is a central issue in cell biology. The forces acting on intracellular vesicles (endosomes) can be assessed in living cells by using a combination of active and passive microrheology.

**Methodology/Principal Findings:**

This dual approach is based on endosome labeling with magnetic nanoparticles. The resulting magnetic endosomes act both as probes that can be manipulated with external magnetic fields to infer the viscoelastic modulus of their surrounding microenvironment, and as biological vehicles that are trafficked along the microtubule network by means of forces generated by molecular motors. The intracellular viscoelastic modulus exhibits power law dependence with frequency, which is microtubule and actin-dependent. The mean square displacements of endosomes do not follow the predictions of the fluctuation-dissipation theorem, which offers evidence for active force generation. Microtubule disruption brings the intracellular medium closer to thermal equilibrium: active forces acting on the endosomes depend on microtubule-associated motors. The power spectra of these active forces, deduced through the use of a generalized Langevin equation, show a power law decrease with frequency and reveal an actin-dependent persistence of the force with time. Experimental spectra have been reproduced by a simple model consisting in a series of force steps power-law distributed in time. This model enlightens the role of the cytoskeleton dependent force exerted on endosomes to perform intracellular trafficking.

**Conclusions/Significance:**

In this work, the influence of cytoskeleton components and molecular motors on intracellular viscoelasticity and transport is addressed. The use of an original probe, the magnetic endosome, allows retrieving the power spectrum of active forces on these organelles thanks to interrelated active and passive measures.

Finally a computational model gives estimates of the force itself and hence of the number of the motors pulling on endosomes.

## Introduction

Regulation of intracellular trafficking is a central issue in cell biology. Extracellular material entering the cell is delivered to a series of vesicular compartments (endosomes), which move along microtubular tracks for intracellular processing. This directional transport is mediated by members of the kinesin and dynein motor families, which convert the chemical energy of ATP hydrolysis into movement. Measuring the forces developed by these microtubule-associated motors may be important for understanding intracellular trafficking. In vitro studies using optical traps and single-molecule fluorescence imaging have provided insights into the driving force exerted by a single molecular motor moving along a microtubule track. Both kinesin and dynein move in 8-nm steps, with a load of up to 7–8 pN before detaching from the microtubule [1], [2], [3], [4].

Although considerable efforts have been made to determine the velocity of single molecular motors and vesicular endosomes inside living cells [5], [6], little is known about the force required to move an endosome along a microtubule in vivo, probably owing to difficulties in manipulating organelles in a controlled manner within a living cell.

Here we demonstrate that this issue can be addressed by combined active and passive intracellular microrheological studies of endosomes.

Microrheology has recently emerged as a useful tool for exploring local mechanical properties of complex systems at microscopic scales. The active approach consists of imposing a controlled stress on a micron-sized probe and measuring its response. The passive approach consists of tracking the spontaneous motion of a probe. While active manipulation of beads attached to the cell surface by means of optical or magnetic tweezers has been extensively used to explore the dynamic behaviour of the cortical cytoskeleton [7], [8], [9], active intracellular microrheology is still in its infancy. Probes are introduced by exploiting natural cellular uptake processes (usually phagocytosis [10], [11], [12]). By contrast, passive microrheology uses a broad range of probes (phagocytosed beads [13], [14], micro-injected particles [15], [16], endogeneous granules [17]), to explore the local physical environment in specific intracellular regions. Indeed, in a system at equilibrium, the fluctuation-dissipation theorem (FDT) unifies the passive and active approaches, by relating the complex shear modulus to the spectrum of thermal fluctuations. However, the use of the FDT is tricky in living cells, which are non-equilibrium soft materials that consume and dissipate energy in their surroundings [18], [19], [20], [21]. In particular, endosome trafficking along microtubular tracks involves the conversion of chemical energy derived from ATP hydrolysis into mechanical work performed by kinesin and dynein molecular motors, in violation of the equilibrium hypothesis of the FDT. Conversely, the use of both active and passive measurements should allow this motor activity to be assessed in terms of the active force generated during trafficking. To our knowledge, this issue has not yet been addressed by combining active and passive studies of intracellular endosomes. For this purpose, we labeled endosomes with magnetic nanoparticles internalized through the endocytosis pathway, and we both manipulated the resulting magnetic endosomes with an external magnetic field and monitored their spontaneous displacements.

## Results

### Magnetic endosomes as intracellular probes

**Figure 1:**
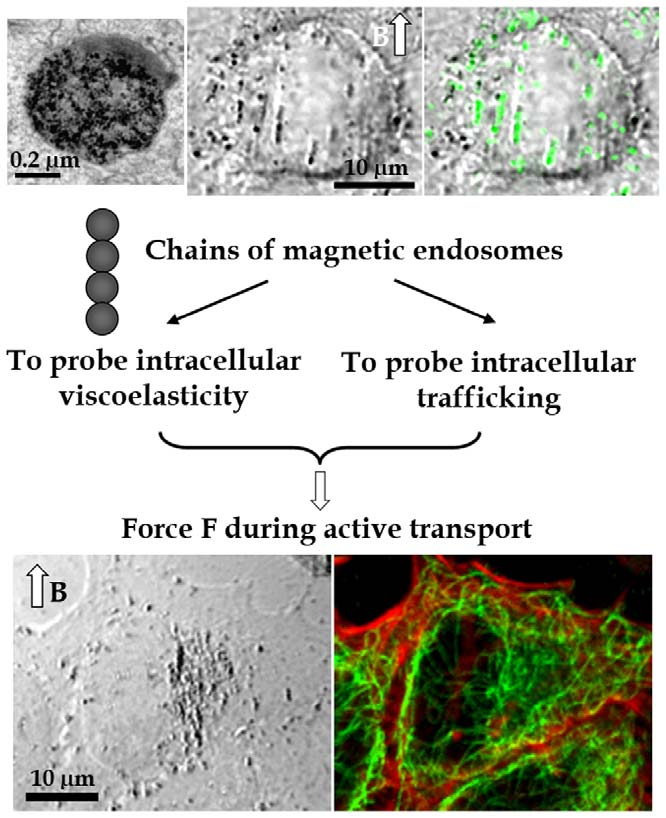
Magnetic endosomes, probes inside living cells. Magnetic nanoparticles (black points on the electron micrograph, top left) concentrate within intracellular endosomes about 0.6 µm in diameter. Each endosome acquires a magnetic moment when submitted to a magnetic field B, resulting in the formation of chains within the cytoplasm, due to dipole-dipole interactions (transmission image, middle top). These chains of magnetic endosomes colocalize with LAMP1 (in green on the superimposed fluorescence image, top right), a marker of late endosomes. The chains are embedded within a dense cytoskeleton network composed of actin filaments (in red on the fluorescence image, bottom right) and microtubules (in green). Chains of endosomes are therefore both biological compartments subject to intracellular trafficking and magnetic probes that can be manipulated by applying an external magnetic field.

Anionic magnetic nanoparticles are conveyed along the endocytosis pathway and are delivered into preexisting endosomal compartments. Internalisation of magnetic nanoparticles is simple to achieve and does not affect normal cellular activities [22]. Using PC3 tumor cells, we found that the nanoparticles concentrated in 0.6-µm-diameter endosomes (5×10^4^ nanoparticles per endosome, Fig. 1). When submitted to an external magnetic field, individual endosomes acquire a magnetic moment and interact with each other to form small cohesive chains consisting of two to eight endosomes (Fig. 1). These chains were then manipulated by applying an external magnetic field. It is noteworthy that the chains colocalized with the late endosome marker LAMP1 (Fig. 1), further demonstrating their endosomal nature. Immersed in the cytoskeleton network (Fig. 1), they should be submitted to driving forces and be shuttled along microtubules.

### Determination of the intracellular viscoelasticity (active microrheology)

**Figure 2:**
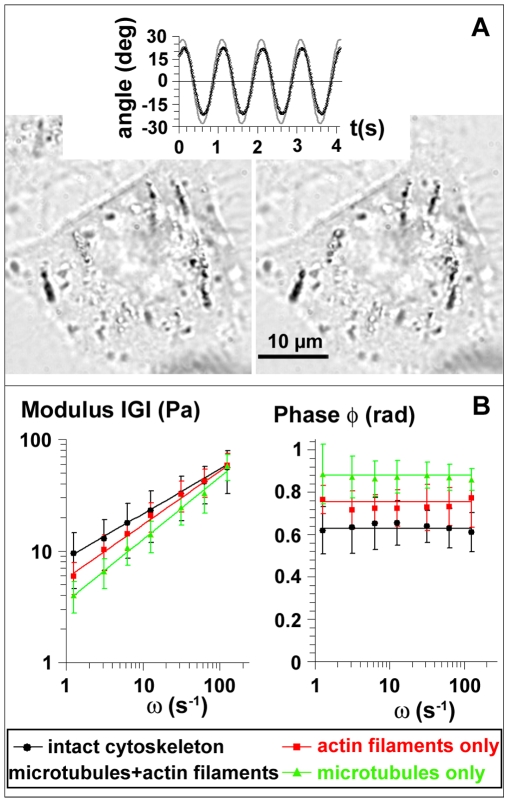
Viscoelasticity of the endosomes microenvironment. (A): A spatially oscillating magnetic field makes chains of endosome oscillate (pictures of chains at their maximum angles for an oscillation of 1 Hz). The angle of each chain (symbols in the upper plot) was monitored by image analysis and compared with the angle of the magnetic field (plain line). (B): The modulus |G(ω)| and phase φ(ω) are represented as a function of frequency for cells with an intact cytoskeleton (black points) and in cells whose microtubules (red, actin filaments only) or actin filaments (green, microtubules only) have been disrupted.

We characterized the mechanical response of the cell interior, and especially the viscoelasticity of the endosomes surrounding, by imposing oscillations to chains of magnetic endosomes. The complex modulus 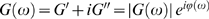 includes both the elastic response (G′) and the viscous response (G″) of the medium. It is related to the ratio θ_o_/β_o_ between the angular amplitude of the chains and the amplitude of the oscillating magnetic field and to the temporal phase lag between the chain oscillations and field oscillations (see Materials and Methods). To assess the influence of cytoskeleton components on the intracellular rheology, cells were treated separately with two cytoskeleton-disrupting drugs. The chains motion was analyzed in cells with an intact cytoskeleton (120 chains), in cells with disrupted microtubules (57 chains) and in cells with disrupted actin filaments (47 chains). In all cases the calculated complex modulus does not depend on the number of endosomes per chain, i.e. on the probe size. Furthermore, it is independent on the magnetic field angular amplitude, ensuring the linearity of the measurement. In cells with an intact cytoskeleton, the frequency dependence of the mean modulus |G| is well represented by a power law |G(ω)| = G_o_ω^α^ with exponent α = 0.40±0.01, and G_o_ = 8.6±2.6 Pa (Fig. 2). |G| exhibits the same power-law behaviour with exponent α = 0.49±0.01 and G_o_ = 5.7±1.8 Pa when microtubules are disrupted (actin filaments only), and with α = 0.56±0.01 and G_o_ = 3.5±1.6 Pa when actin filaments are disrupted (microtubules only) (Fig. 2). The phase φ remains constant in all conditions, and verifies the relation 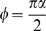 (Fig. 2), meaning that the complex modulus itself follows power-law behaviour 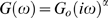.

### Magnetic endosomes dynamics (passive microrheology)

**Figure 3:**
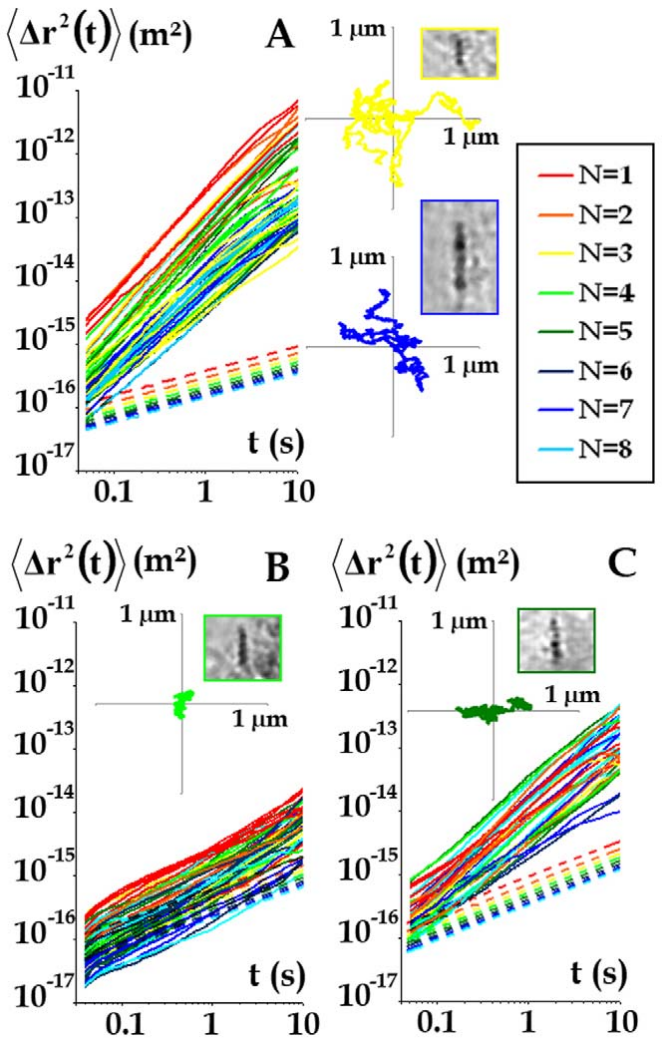
Probes fluctuations. The mean square displacement 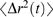 is colour-coded according to the number of endosomes, N, in the chain, and plotted against time (solid lines), in cells with an intact cytoskeleton (*A*) and in cells with actin filaments only (B) or microtubules only (C). The dotted lines correspond to the mean square displacement 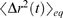 calculated using the FDT, as if the system was at equilibrium. Typical trajectories are presented in the insets, together with a view of the corresponding chain.

In a second step, we measured the spontaneous fluctuations of magnetic endosome positions and calculated their temporal mean-square displacements 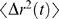. Experimentally, chains of endosomes were tracked every 0.01 s (Fig. 3, insets) in cells with an intact cytoskeleton (54 chains), in cells with disrupted microtubules (55 chains) and in cells with disrupted actin filaments (36 chains), and the corresponding mean square displacements 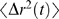 were computed from the tracks. They all display power law behaviour with time: 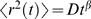 (Fig. 3). Exponent β was found to be larger than 1 (β = 1.3±0.1) in cells with an intact cytoskeleton (superdiffusive motion). The exponent fell slightly when actin filaments were disrupted (β = 1.2±0.1) and more markedly when microtubules were disrupted (β = 0.8±0.2) (subdiffusive motion).

To compare the experimental 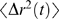 to its value in an equilibrium situation 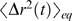, we use the fluctuation-dissipation theorem (FDT), which relates, at thermal equilibrium, the Fourier transform, 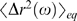 of 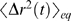 to the macroscopic viscoelastic modulus G(ω) (see Materials and Methods, Eq. 1). The so obtained expected in-equilibrium 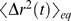 are represented by the dotted lines in Fig. 3. It corresponds to thermal motion in a viscoelastic medium and displays subdiffusive behaviour, 
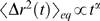
, with the same exponent α as in G(ω). In control cells, we observe a strong mismatch between algebraic values of the measured 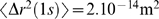, compared to 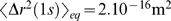 for the in-equilibrium situation. Also, β = 1.3 is found much larger than α = 0.4. This demonstrates direct violation of the FDT.

By contrast, in cells with disrupted microtubules, the measured 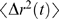 approximately matches the values 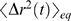 predicted by FDT. This directly demonstrates that the active non-thermal driving forces are generated by microtubule-associated motors.

Here we limited the analysis to the mean square displacement 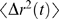, as it is sufficient to derive the power spectra of forces (according to Eq. 2). However, it has to be noted that further explorations of the spontaneous probes motions could be performed, using in particular a segmentation analysis, which may lead to new data on the force experienced during motion [23].

### Power spectra of intracellular forces acting on endosomes

**Figure 4:**
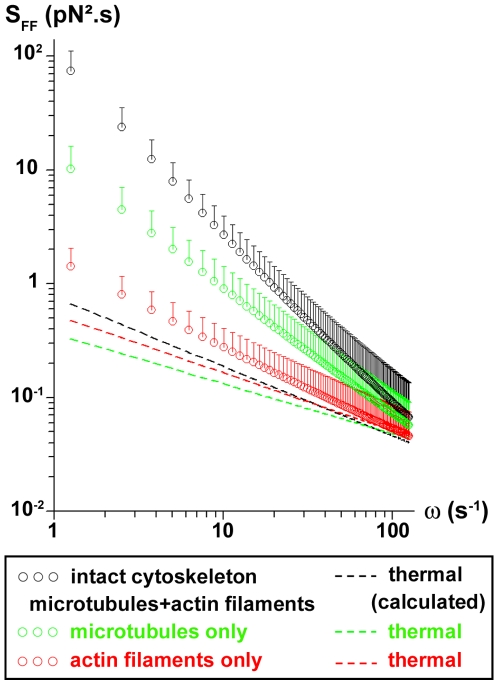
Power spectra of intracellular forces on endosomes. Force power spectra are averaged over all the probes tracked, inside cells with an intact cytoskeleton (black symbols), or with microtubules (green symbols) or actin filaments (red symbols) only. The dotted lines represent the spectra of the thermal forces in each case.

Data from active and passive experiments (performed with the same probes, in the same intracellular location) were then combined in a Langevin-type approach to measure the forces responsible for the active displacement of endosomes (see Methods). According to Eq. 2, the force fluctuation spectrum 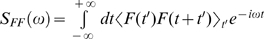 is related to the viscoelastic modulus and to the real part of Fourier transform <Δr^2^(ω)> of the probe's mean square displacement: 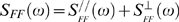, with 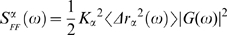, 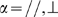. For all the tracked probes, S_FF_(ω) varies with frequency as S_FF_(ω) = Aω^−γ^. Interestingly, both the intensity of the force power spectrum A and the exponent γ are found uncorrelated with the number of endosomes in the chains probe. This simply means that the force on a chain is generated on a single endosome, driving the whole chain into movement. We find γ = 1.5±0.2 in cells with an intact cytoskeleton, which verifies 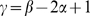 as calculated from Eq. 2. When actin filaments are disrupted, we obtain γ = 1±0.15. Microtubule disruption leads to γ = 0.8±0.2. Fig. 4 shows the force fluctuation spectra average over all the tracked probes, in normal conditions and inside cells with only microtubules (actin filaments disrupted) or only actin filaments (microtubules disrupted). The expected spectrum at equilibrium, which corresponds to the thermal forces fluctuation spectrum, is represented in each case. The measured spectra when microtubules are present (intact cytoskeleton or only microtubules) strongly mismatch the thermal spectra, demonstrating that the major component to the force acting on the endosomes is the motors-mediated active force. By contrast, when microtubules are disrupted (only actin filaments), the measured spectrum comes closer to the thermal spectrum, and thermal forces get significant for the movement.

### Model

In the presence of microtubules, the force fluctuation spectra differ from the equilibrium ones: it is the signature of active forces exerted on the endosomes. We propose a simple model based on the distributions of step force durations to interpret the experimental spectrum and its power law behavior. We first hypothesize that the magnetic endosomes probes are equipped with molecular motors that can generate movement along a microtubule, in either plus and minus end directions. This is justified by the demonstrated role of the microtubules in the force generation. Besides it was demonstrated in a previous in vitro study that purified magnetic endosomes could move on reconstituted microtubules networks, with two kinesins responsible for the movement, kinesin 2 toward the plus end and kinesin 14 toward the minus end [24].

**Figure 5:**
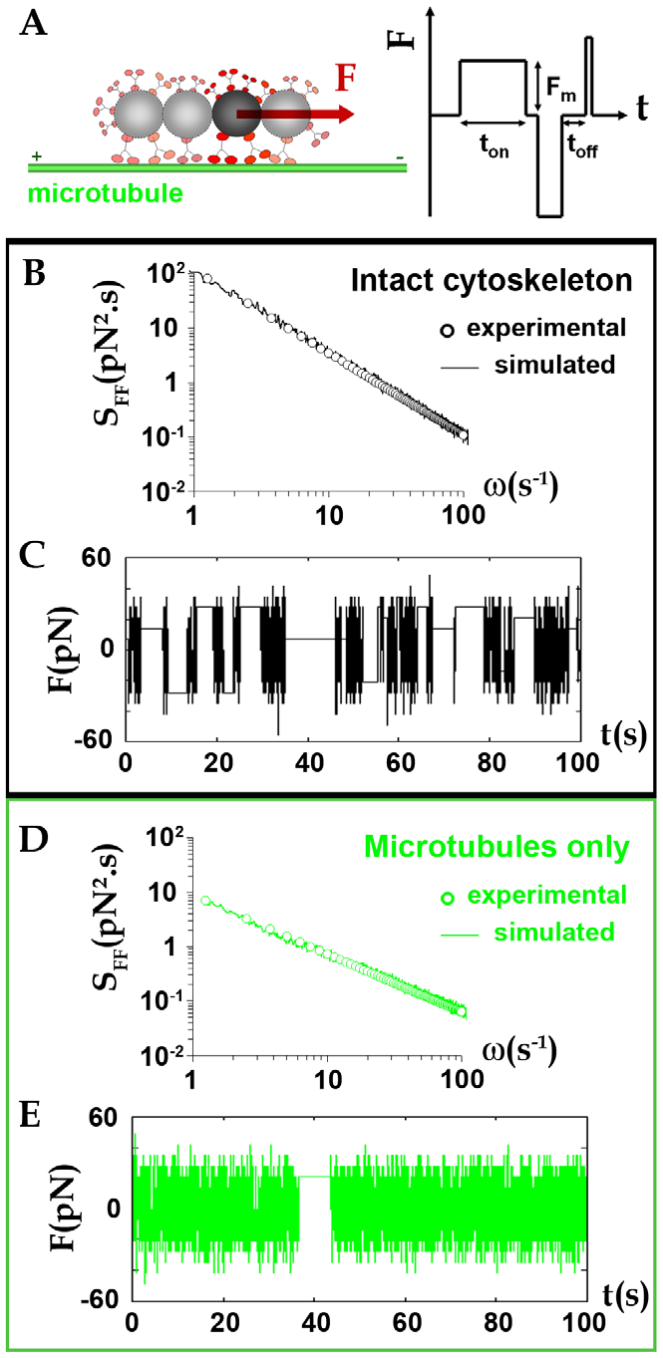
Modeling the active forces. (A): The chains of endosomes probes (with varying number of endosomes in it) can be seen as actively driven over the cytoplasm under the effect of a force F(t) created by microtubule dependent molecular motors, generating movement along one microtubule in both directions (depending on the type of motor). (B): Superposition of an experimental spectrum obtained in cell with intact cytoskeleton (open symbols) S_FF_ = Aω^−γ^ (A = 10^−22^ N^2^s, γ = 1.5) with the spectrum simulated using the model proposed (line), with parameters (F_mean_ = 22 pN, x = 1.5). (C): Corresponding time dependent force F(t). (D): In cell with microtubules only, experimental spectrum (open symbols) S_FF_ = Aω^−γ^ (A = 10^−21^, γ = 1.1) superimposed with simulated spectrum (F_mean_ = 20 pN, x = 1.9). (E): Corresponding time dependent force F(t).

We introduce a time dependent force F(t), described as the resultant of successive positive or negative force steps generated by an assembly of molecular motors acting on the endosomes (Fig. 5A). These steps are specifically distributed in time, amplitude and duration. Here we assume that the duration t_on_ of the force steps is power-law distributed as 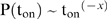. The force F(t) is therefore characterized by two control parameters: the average force amplitude during the endosome trajectory F_mean_ and the exponent x of the power law distribution (see Materials and Methods section for details). F_mean_ is proportional to the average number n_mot_ of motors able to work together and x is an indicative of the persistence of the force generated: the larger the exponent x, the less frequent are long duration t_on_.

Fig. 5B shows a simulated spectrum (plain line) computed to match one (open circles) of the experimental spectra obtained in a cell with intact cytoskeleton, with parameters (F_mean_ = 22 pN, x = 1.5). A typical time sequence for the corresponding force F(t) is illustrated in Fig. 5C. This simulated force temporarily reaches 55 pN and can be applied during times that can last more than 10 s. In Fig. 5D, an experimental spectrum obtain in cell with only microtubules is represented together with its simulated spectrum (F_mean_ = 20 pN, x = 1.9 – plain line) and a time sequence for F(t) is shown in Fig. 5E. Again, the force attains up to 50 pN, with an average at 20 pN, but the duration of force steps never exceed a few seconds.

**Figure 6:**
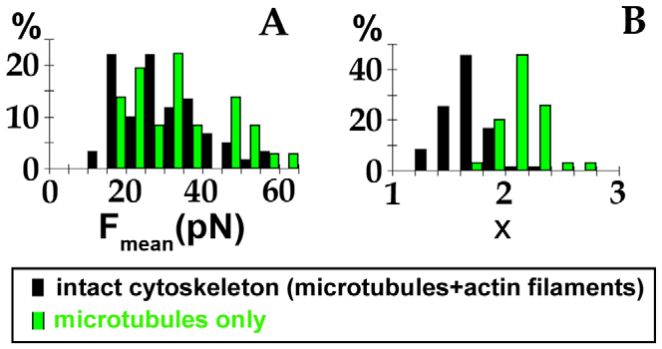
Parameters computed from the force model. Histograms of the parameters F_mean_ (A) and x (B), respectively corresponding to the mean absolute force over one trajectory and to the exponent of the distribution P(t_on_)∼t_on_^(−x)^ of the duration of single force steps (see Model section). F_mean_ is log-normally distributed and x is normally distributed over all the probes tested (% of probes).

We adjusted all the experimental spectra with this model, for control cells with both actin filaments and microtubules (intact cytoskeleton) and in cells with microtubules only. The histograms of mean force amplitude applied during a single endosome trajectory F_mean_ and of the exponents x are shown in Figs. 6A and 6B. The average force F_mean_ is log-normally distributed around 26 pN for both cells with intact cytoskeleton or with microtubules only. One must keep in mind that F_mean_ corresponds to the average amplitude of the instantaneous force F acting along a single probe trajectory. The simulations also give access to the maximum values attained by F, which can attain up to 140 pN. The exponent x is normally distributed around its mean value. Besides, a difference was revealed between the x values retrieved for cells with intact cytoskeleton (x∼1.5) and the ones obtained for cells with only microtubules and no more actin filaments (x∼2). The increase of the exponent when the actin cytoskeleton is disrupted means that long duration steps are less likely to occur.

## Discussion

### Power-law intracellular microrheology

Chains of magnetic endosomes emerged as experimental probes of the intracellular viscoelasticity and allowed retrieving the intracellular complex shear modulus G(ω).

G(ω) follows power-law behaviour: 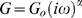. This demonstrates that intracellular microrheology is not tied to a particular relaxation time but is governed by multiscale processes involving a distribution of relaxation times. This is in agreement with previous studies of cortex microrheology, using extracellular probes interacting with the cell membrane, which implied power-law cell mechanical moduli with an exponent close to 0.2 [7]. By contrast, only two studies have reported power-law viscoelastic moduli obtained with intracellular probes, but with specialized cells (phagocyting amoeba [25], and migrating fibroblasts containing lipid granules [26]). The exponent was found about 0.5 in both cases. Passive intracellular microrheology experiments with ATP-depleted cells, which can be considered close to thermal equilibrium, lead to similar behaviour [27], with an exponent (around 0.3) larger than the values obtained in cortical experiments. Here, not only do we show that such power-law behaviour applies to a generic mammalian tumour cell model but we also identify for the first time the roles of actin filaments and microtubules. Suppression of one of these two major cytoskeleton components increased the measured exponent from 0.4 for cells with an intact cytoskeleton to 0.5 for cells with microtubules disrupted and 0.6 for cells with disrupted actin filaments. The exponent α quantifies the extent of fluidlike versus solidlike behaviour (α = 0 for a purely elastic material, α = 1 for a viscous fluid). Disruption of microtubules or actin filaments leads to a more fluid cytoplasm: both structures, but especially actin filaments, contribute to the elasticity of the intracellular cytoskeleton.

### Departure from the fluctuation-dissipation theorem with endosomes probes

If the fluctuation-dissipation theorem (thermodynamic equilibrium) was to apply to this complex intracellular system with power-law viscoelastic behavior, then the movement of the endosomal probes should be subdiffusive, with an exponent equal to the one of the viscoelastic modulus (β = α). However, in cells with an intact cytoskeleton, and even in cells lacking actin filaments, the movement is largely superdiffusive, with an exponent β∼1.3.

To quantify the deviation from equilibrium in evolving systems, it has been proposed [25], [28] to replace the temperature in Eq. 1 (FDT) by a frequency-dependent effective temperature, T_eff_(ω). T_eff_ is then the ratio between the experimental mean square displacement (plain lines in Fig. 3) to the one predicted by the FDT (dotted lines in Fig. 3). The deviation from the bath temperature is almost 1000 times that of the bath at 1 s^−1^. In contrast, microtubule disruption brings the system close to equilibrium, with an effective temperature barely 10 times that of the bath at 1 s^−1^. These early measurements clearly demonstrate that the cell interior deviates from thermodynamic equilibrium when using an endosomal probe. Such a deviation from equilibrium has previously been observed in the cytoplasm of highly motile amoebae in experiments with phagosomal probes [25], and in a system using an active actin gel recomposed outside the cell and micrometric bead probes [20].

In summary of this section, we have measured the deviation from equilibrium of the intracellular medium in a generic model of malignant human cells and underlined that the concept of passive microrheology cannot be directly apply to cells. Besides, we have demonstrated that, with probes introduced by a cellular uptake processes, a direct application of the passive microrheology approach (and in particular the FDT formulation) leads to incorrect results for the shear complex modulus *G*(ω).

Additionally, this work illustrates the role of the microtubules to create the out of equilibrium situation. In particular, we demonstrate that microtubules disruption brings the system close to the equilibrium situation. Microtubules appear therefore as the main support of active non-thermal driving forces. Considering the endosomal nature of the probes, it seems justified to assume that these forces are mediated by microtubule-associated motors.

Here the magnetic nanoparticles, mediators for the magnetic oscillations, were engulfed inside the cells into membrane delimited vesicles, and then delivered into pre-existing endosomes, again membrane delimited. The active force responsible for the out of equilibrium situation is created by molecular motors present at the endosomal membrane. If the probes were to be microinjected and not engulfed, they may not be actively interacting with the cell machinery, and behave as passive probes which spontaneous motions can be used to infer the mechanical properties of their surroundings. It has been indeed recently demonstrated that thermal forces only drive the motion of 100 nm-diameter polystyrene nanoparticles probes directly injected into the cytoplasm of cells [29].

### Power spectrum of intracellular forces acting on endosomes

As a second step, we investigated the motor forces acting on the endosomal probe, which dominate over thermal forces.

The power spectrum S_FF_(ω) varies with frequency as ω^−γ^. Inside cells with an intact cytoskeleton, we found an exponent γ∼1.5, in the explored temporal region (0.05–5s), which strongly mismatches the one expected at thermal equilibrium, γ^eq^ = 0.6, deduced from the measured intracellular viscoelastic modulus. A few years ago Lau et al. [18] proposed a force spectrum S_FF_(ω) proportional to 1/ω^2^, which corresponds to an exponent γ = 2. They suggested that a linear growth with time of the force autocorrelation function, attributed to very slow changes in the intracellular constraint, could generate such behaviour. However, this interpretation was referring to a comparison between active and passive measurements obtained with different probes and at very different locations (passive probes: submicrometric endogenous granules effectively immersed in the cytoplasm; active probes: micrometric beads attached to the membrane). Since this time, dual passive-active measurements have been made with a unique probe (a bead attached to the membrane), yielding an exponent γ between 1.7 and 2.5 (force spectrum between 1/ω^1.7^ and 1/ω^2.5^) [19], [30]. A force spectrum in 1/ω^2^ has also been found in an in vitro active gel made of crosslinked actin with myosins added [20]. This 1/ω^2^ behaviour was recently interpreted as the result of series of force square steps, having random durations around an average value τ, and produced by assemblies of molecular motors [31]. This generates a 1/ω^2^ spectrum only in a frequency range such that ωτ>>1.

### Amplitude and temporal distribution of force steps on endosomes

Finally, we developed a simple computational model to adjust the experimental force spectra. Active force generation is subjected to the presence of the microtubule network. The force modelling was then performed solely on cells with microtubules, with or without actin filaments. If the microtubules were to be disrupted, one should envisage thermal forces, which are not logically described by a series of force steps as proposed here for active forces.

The average force acting during the endosomes trafficking, F_mean_, calculated from the simulations, is distributed around 26 pN for both cells with intact cytoskeleton or with actin filaments disrupted (Fig. 6A). This 26 pN value is in the same order of magnitude as the stall force developed by a single kinesin (7 pN). More precisely, we can estimate from the simulations that, on average, about 3 motors are working together to pull on the endosomes. While F_mean_ corresponds to the average amplitude of the force F acting all along a single probe trajectory, the instantaneous force F can reach maximum values up to 140 pN (about n = 20 motors). Interestingly, we were also able to experimentally measure the instantaneous force developed on a single endosome. Indeed, we observed that endosome chains happen sometimes to dissociate as they move. This reveals that active forces exerted on the endosomes are able to overcome, at a given time, the cohesive magnetic dipolar force linking the endosome to the chain. To detach a single endosome from a chain, the instantaneous force F_inst_ must counterbalance the cohesive magnetic dipolar force F_d_ that links the endosome to its neighbour: 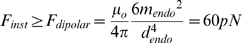. This value is consistent with the amplitude of the fluctuating forces used for the simulations. Therefore, simulations and observations consistently show that molecular motors are strong enough to develop important instantaneous forces, involving more than 10 motors, which transiently cooperate [32], [33].

The simulations provide another interesting finding concerning the maximum duration t_on_ of application of the instantaneous force (related to the exponent x values). Inside cells with an intact cytoskeleton, the existence of long duration force steps (up to 10 s) is demonstrated, meaning that several motors must cooperate together in a processing way. On the contrary, when the actin cytoskeleton is disrupted, long duration steps (more than a few s) become more unlikely: the processivity of the motors decreases. However, the instantaneous amplitude of the force generated on the endosomes is not altered by the disruption of the actin filaments. Actually, the involvement of actin in endosome and lysosome dynamics was only recently recognized, and it is only in recent years that the myosins participating in this dynamic have been identified (especially myosin 1b bound on endosomes and lysosomes). Moreover, several cases of actin-mediated propulsion of endocytosis vesicles have been described, both in yeast cells [34] and in mammalian cells [35], [36], [37]. We recently showed that magnetic endosomes carry myosins 1b and VI but do not move along actin filaments in vitro [24]. Magnetic endosomes were themselves capable of inducing actin polymerization in vitro, possessing proteins involved in signaling pathways that regulate actin dynamics; in vivo, bundles of actin filaments were observed close to the magnetic endosomes, with a structure resembling that of actin comets observed on endosomes [38].

We therefore demonstrate here that, while microtubules form the rails that are essential for active transport of endosomes, actin filaments participate to stabilize the movement.

### Conclusion

This study focuses on the force generation on endosomes probes travelling along microtubules. The key findings are multiple.

- A power-law rheological behaviour is found for the intracellular microenvironment of endosomes. We have shown that selective disruption of the actin network or of the microtubules differently affects the power-law response. Such power law behaviour has been rarely investigated with active measurements inside living cell and was never documented as a function of cytoskeleton components.
- The mean square displacements values show a strong departure from the predictions of the fluctuation-dissipation theorem. Besides, the active forces responsible for this out of equilibrium situation are mainly associated with the existence of microtubules.
- Finally, we have retrieved the power spectrum of active forces pulling on endosomes and developed a model taking into account the distribution of the duration of step forces acting on endosomes. The average amplitude of the corresponding force, in the range of tenth of pN, is of the order of the force developed by an individual kinesin.

## Materials and Methods

### Magnetic labelling of intracellular endosomes

A generic tumour cell model (PC3 human prostatic adenocarcinoma cells, ATCC) cultured in DMEM medium supplemented with glutamine, fetal bovine serum and penicillin/streptomycin was labelled with anionic magnetic nanoparticles. Nanoparticles (synthesized at LI2C, UMR 7612 CNRS and University Pierre et Marie Curie, Paris, France) are made of a metallic core of cobalt ferrite, with diameter 8 nm, and bear negative surface charges due to carboxylate groups complexed on their surface, ensuring their stability in aqueous solution. Magnetic labeling of the tumour cells was performed by adding to the cell medium (supplemented with 5 mM citrate) a filter-sterilised suspension of magnetic nanoparticles at an extracellular iron concentration of 5 mM, for 30 min. The cells were then washed and incubated for two hours to permit nanoparticle internalisation and confinement within late endosomes. The intracellular location of the nanoparticles was then checked by using transmission electron microscopy (performed at INRA, Jouy en Josas, France) and immunofluorescence with a mouse anti-Lamp-1 antibody and confocal microscopy.

### Intracellular active magnetic endosome microrheology

Chains of magnetic endosomes were aligned along a homogeneous horizontal magnetic field of 76 mT, generated by two permanent magnets, and were made to oscillate by applying an additional field generated by a pair of perpendicular coils (each 6mH inductance) supplied with a sinusoidal current. A home made amplifier allows delivering current up to 4A to the coils. The applied voltage has to be tuned with frequency so that the amplifier delivers a given current (1.3V from 0.2Hz to 1Hz, 1.4V, 1.7V and 2.5V at 5, 10, 20 Hz respectively, to deliver a 4A current). For each frequency, the phase-lag between the current and the voltage was carefully measured (e.g 4ms at 0.5Hz, 7.7ms at 20Hz). The magnetic field created increases with the current circulating in the coils (B(1A) = 11 mT, B(2A) = 21 mT, B(4A) = 42 mT). Finally, the magnetic field is perfectly calibrated, with constant amplitude for every tested frequency, and with its known phase chosen as reference for the probes subsequent oscillations. This resulting spatially oscillating magnetic field is described by its rotation angle β = β_o_e^iωt^. In response, the chains of magnetic endosomes oscillation is described by the angle θ = θ_o_e^i(ωt-δ)^. The angle of each chain is monitored by videomicroscopy (ultra-fast camera sampling up to 1000 frames per second) and image analysis, and compared with the angle of the magnetic field. The complex shear modulus G*(ω) = G′(ω)+iG″(ω) = |G(ω)|e^iφ(ω)^, can then be derived from the ratio of amplitude θ_o_/β_o_ and from the phase lag δ of a chain as: G′ = Γ_o_/κV{β_o_/θ_o_*cos(δ)−1} and G″ = Γ_o_/κV*β_o_/θ_o_*sin(δ). V is the chain volume. Γ_o_ is a magnetic factor calculated from the magnetic interaction of one magnetic endosome with its close neighbours: Γ_o_ = 3µ_o_m^2^/4π*N^2^/d_endo_^3^, where m is the endosome magnetic moment m = 3.7×10^−15^A.m^2^, N, the number of endosomes in the chain and d_endo_ = 0.6 µm the endosome diameter. κ is a geometric factor which was calibrated as a function of the number of magnetic endosomes per chain N in a Maxwellian fluid: κ = 2N^2^/(ln(N/2)+2.4/N) [39].

To assess the influence of cytoskeleton components on the intracellular complex shear modulus, cells were treated separately with two cytoskeleton-disrupting drugs, namely latrunculin A (10 min, 1 µM) and nocodazole (30 min, 10 µM).

### Intracellular passive endosome microrheology

The passive approach measures the spontaneous motion of probes in the absence of external driving forces. Chains of endosomes as well as single endosomes were tracked in cells at 60 frames per second, using ImageJ 1.41 (Wayne Rasband National Institude of Health, USA) and a home made particle tracker plug-in (source available at http://www.msc.univ-paris-diderot.fr/~olivier/plugins/). The mean-square displacement of each track was computed according to:


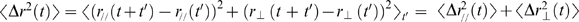
, where 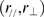 is the position of the chain center at time t, along or perpendicular to the chain direction. The values are averaged over time t'.

When passive microrheology is performed in a viscoelastic medium at thermal equilibrium, i.e. with no additional energy supply, the mean square displacement analysis provides information on the rheology of the material, and yields the frequency-dependent complex shear modulus. A decade ago, Mason and Weitz proposed an elegant scheme that links the shear modulus to the Laplace transformed mean square displacement which can be seen as a derivation of the fluctuation-dissipation theorem. If this approach was valid for passive endosome microrheology, then the Fourier transform real part 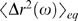
can be calculated from G(ω), according to:
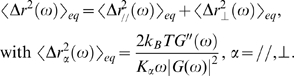
(1)

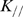
 and 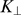 are geometric factors expressed in meters. For a sphere of diameter a, 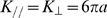. For a chain of N spheres, 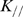 and 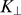 were calibrated in a Newtonian fluid, for N varying from 2 to 8, and were found to follow phenomenological laws: 
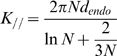
 and 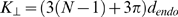, where d_endo_ = 0.6µm is the endosome diameter.

### Use of generalized Langevin formalism to derive the force exerted on endosomes

Whether or not the system is at equilibrium, the spontaneous motion of an intracellular probe is described by a generalized Langevin equation:
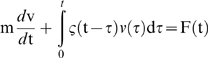
, in which v(t) is the probe velocity, ζ(t) a delayed friction function that takes into account the viscoelastic properties, and F(t) the force acting on the probe, including both the contributions of thermal Brownian forces, driving forces generated by the molecular motors, and external forces. A generalized Stokes equation links the Fourier transform of ζ(t) to G(ω): 
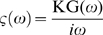
. K is the same geometric factor as above. Fourier transform of the Langevin equation, combined with the Wiener-Khinchin theorem, under the assumption that inertia is negligible for micron-sized endosomes, allows one to relate the force fluctuation spectrum (autocorrelation function) 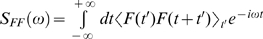 to the viscoelastic modulus and to the real part Fourier transform <Δr^2^(ω)> of the probe's mean square displacement:
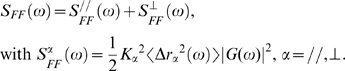
(2)This relation is always valid, whether the system is at equilibrium or not.

At equilibrium, the power spectrum of thermal forces is obtained by combining Eq. (1) and Eq. (2): 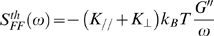.

To check the validity of Eq. (2) for the measure of the thermal force fluctuation spectrum (for the in equilibrium situation), we performed the two microrheological techniques in a linear viscous fluid (a mixture of glycerol 98% in water at 25°C) with chains of 1µm diameter magnetic beads (with magnetic moment 10^−14^ A.m^2^). Both the active and passive measurements were conducted with number N of beads in the chains probes varying from 2 to 5 (with an additional measure with N = 1 in the passive case). The active determination of the complex shear modulus G*(ω) = G′(ω)+iG″(ω) demonstrated that G′ is negligible compared to G″ (with a ratio G′/G″ in between 10^−3^ and 10^−2^ for all the measurements) and that G″ increased linearly with the frequency : G″ = ηω. η did not depend on the number of beads in the probed chains, and was distributed as η = 619±24 mPa.s. The experimental mean square displacements computed from the tracks of the chains wrote 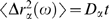, with 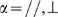 (e.g., for a 2 beads chain, 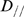 = (1.2±0.3).10^−15^m^2^.s^−1^/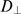 = (1.1±0.4).10^−15^m^2^.s^−1^ and for a 5 beads chain, 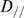 = (8.3±1).10^−16^m^2^.s^−1^/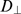 = (8.2±1.2).10^−16^m^2^.s^−1^). The experimental 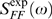 were deduced from equation (2), and did not vary with frequency (e.g. for a 2 beads chain, 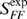 = (1.5±0.2).10^−25^N^2^.s and for a 5 beads chain, 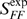 = (2.5±0.3).10^−25^N^2^.s). The thermal forces power spectrum 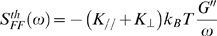 were directly calculated from G″ (e.g. for a 2 beads chain, 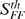 = 1.3.10^−25^N^2^.s and for a 5 beads chain, 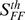 = 2.1.10^−25^N^2^.s). For all the measurements, the ratio 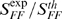 is found in between 0.9 and 1.1, justifying the use of equation (2) to infer the force fluctuation spectrum.

### Modelisation of active forces

The model force proposed consists in a series of force steps, positive and negative, with varying duration, t_on_. The key parameter is the temporal distribution of such square pulses. The dwell time, t_off_, which is the time between two adjacent force square pulses, is assumed to have an exponential distribution, as shown in references [2], [40] : 
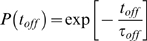
 with τ_off_ = 30ms. This distribution means that the attachment to the microtubule is independent of the duration of the motor activity.

For the distribution of the duration of force steps, we assume that t_on_ varies in a large range of values and we propose a power law distribution for t_on_ : P(t_on_)∼t_on_^(−x)^, with x positive. The hypothesis stands in intermittent pulling of the motors to develop a driving force. It first comes naturally to distribute exponentially the duration of force application [31], [41]. Using such a distribution led to a two-regime in frequency for the force power spectrum : constant at low frequency, and with 1/ω^2^ behaviour at high frequency. Here we identified a single frequency behaviour for the frequency range explored, varying as a power-law, with an exponent inferior to 2. Besides, on the tracked trajectories, no characteristic times emerge, while occasionally events of long step duration occur. This grounded our modeling with a power-law distribution for the force duration P(t_on_) = t_on_^−x^. We have restricted the variations of t_on_ in a physical range between 0.1 ms (the numerical step) and 100s. As a final step, we assume an identical probability for both directions of the force (that is a positive or negative force value), and a variable number of motors pulling together on the endosome probes. So for each pulse we consider a random number n of working motors, normally distributed around an average value n_mot_. The amplitude of each pulse is nF_0_, where F_0_ is taken as the force exerted by a single motor. The numerical parameter that can be extracted from the simulation is then the average force acting on the probe over one trajectory F_mean_ = n_mot_F_0_.

The corresponding power spectrum S_FF_^num^(*ω*) is then easily calculable numerically using fast Fourier transform. For x values up to 2.5, we find a power law behaviour for S_FF_^num^(*ω*) in a large frequency range (2.10^−2^Hz–2.10*^2^*Hz) For each experimental run, we adjust S_FF_^num^(*ω*) to the experimental spectrum S_FF_(*ω*). Thus we can associate, to each tracked trajectory, a time dependent microscopic force F(t) and the two corresponding parameters F_mean_ and x. All this work has been done using MATLAB 7.0 (The Math-Works, Natick, MA).

## References

[1] Svoboda K, Schmidt CF, Schnapp BJ, Block SM (1993). Direct observation of kinesin stepping by optical trapping interferometry.. Nature.

[2] Kojima H, Muto E, Higuchi H, Yanagida T (1997). Mechanics of single kinesin molecules measured by optical trapping nanometry.. Biophys J.

[3] Visscher K, Schnitzer MJ, Block SM (1999). Single kinesin molecules studied with a molecular force clamp.. Nature.

[4] Toba S, Watanabe TM, Yamaguchi-Okimoto L, Toyoshima YY, Higuchi H (2006). Overlapping hand-over-hand mechanism of single molecular motility of cytoplasmic dynein.. Proc Natl Acad Sci USA.

[5] Kural C, Kim H, Syed S, Goshima G, Gelfand VI (2005). Kinesin and dynein move a peroxisome in vivo: a tug-of-war or coordinated movement?. Science.

[6] Nan X, Sims PA, Xie XS (2008). Organelle tracking in a living cell with microsecond time resolution and nanometer spatial precision.. Chem Phys Chem.

[7] Fabry B, Maksym GN, Butler JP, Glogauer M, Navajas D (2001). Scaling the microrheology of living cells.. Phys Rev Lett.

[8] Balland M, Desprat N, Icard D, Féréol S, Asnacios A (2006). Power laws in microrheology experiments on living cells: Comparative analysis and modeling.. Phys Rev E.

[9] Trepat X, Lenormand G, Fredberg JJ (2008). Universality in cell mechanics.. Soft Matter.

[10] Bausch AR, Möller W, Sackmann E (1999). Measurement of local viscoelasticity and forces in living cells by magnetic tweezers.. Biophys J.

[11] Feneberg W, Westphal M, Sackmann E (2001). Dictyostelium cells' cytoplasm as an active viscoplastic body.. Eur Biophys J.

[12] Marion S, Wilhelm C, Voigt H, Bacri JC, Guillén N (2004). Overexpression of myosin IB in living Entamoeba histolytica enhances cytoplasm viscosity and reduces phagocytosis.. J Cell Sci.

[13] Caspi A, Granek R, Elbaum M (2000). Enhanced diffusion in active intracellular transport.. Phys Rev Lett.

[14] Girard KD, Kuo SC, Robinson DN (2006). Dictyostelium myosin II mechanochemistry promotes active behavior of the cortex on long time scales.. Proc Natl Acad Sci USA.

[15] Heidemann SR, Wirtz D (2004). Towards a regional approach to cell mechanics.. Trends Cell Biol.

[16] Guigas G, Kalla C, Weiss M (2007). Probing the nanoscale viscoelasticity of intracellular fluids in living cells.. Biophys J.

[17] Yamada S, Wirtz D, Kuo SC (2000). Mechanics of living cells measured by laser tracking microrheology.. Biophys J.

[18] Lau AWC, Hoffman BD, Davies A, Crocker JC, Lubensky TC (2003). Microrheology, Stress Fluctuations, and Active Behavior of Living Cells.. Phys Rev Lett.

[19] Bursac P, Lenormand G, Fabry B, Oliver M, Weitz DA (2005). Cytoskeletal remodelling and slow dynamics in the living cell.. Nat Mater.

[20] Mizuno D, Tardin C, Schmidt CF, Mackintosh FC (2007). Nonequilibrium mechanics of active cytoskeletal networks.. Science.

[21] Brangwynne CP, Koenderink GH, MacKintosh FC, Weitz DA (2008). Cytoplasmic diffusion: molecular motors mix it up.. J Cell Biol.

[22] Wilhelm C, Gazeau F (2008). Universal cell labelling with anionic magnetic nanoparticles.. Biomaterials.

[23] Masson J-B, Casanova D, Turkcan S, Voisinne G, Popoff MR (2009). Inferring maps of forces inside cell membrane microdomains.. Phys Rev Lett.

[24] Loubéry S, Wilhelm C, Hurbain I, Neveu S, Louvard D (2008). Different microtubule motors move early and late endocytic compartments.. Traffic.

[25] Wilhelm C (2008). Out-of-equilibrium microrheology inside living cells.. Phys Rev Lett.

[26] Yanai M, Butler JP, Suzuki T, Sasaki H, Higuchi H (2004). Regional rheological differences in locomoting neutrophils.. Am J Physiol Cell Physiol.

[27] Hoffman BD, Massiera G, Van Citters KM, Crocker JC (2006). The consensus mechanics of cultured mammalian cells.. Proc Natl Acad Sci USA.

[28] Pottier N (2005). Out of equilibrium Stokes-Einstein relation: determination of the effective temperature of an aging medium.. Physica A.

[29] Hale CM, Sun SX, Wirtz D (2009). Resolving the role of actoymyosin contractility in cell microrheology.. PloS one.

[30] Gallet F, Arcizet D, Bohec P, Richert A (2009). Power spectrum of out-of-equilibrium forces in living cells: amplitude and frequency dependence.. Soft Matter.

[31] Levine AJ, Mackintosh FC (2009). The Mechanics and Fluctuation Spectrum of Active Gels.. J Phys Chem B.

[32] Zeldovich KB, Joanny JF, Prost J (2005). Motor proteins transporting cargos.. Eur Phys J E.

[33] Arcizet D, Meier B, Sackmann E, Radler JO, Heinrich D (2009). Temporal Analysis of Active and Passive Transport in Living Cells.. Phys Rev Lett.

[34] Kaksonen M, Toret CP, Drubin DG (2005). A modular design for the clathrin- and actin-mediated endocytosis machinery.. Cell.

[35] Merrifield CJ, Moss SE, Ballestrem C, Imhof BA, Giese G (1999). Endocytic vesicles move at the tips of actin tails in cultured mast cells.. Nat Cell Biol.

[36] Perrais D, Merrifield CJ (2005). Dynamics of endocytic vesicle creation.. Dev Cell.

[37] Smythe E, Ayscough KR (2006). Actin regulation in endocytosis.. J Cell Sci.

[38] Taunton J (2001). Actin filament nucleation by endosomes, lysosomes and secretory vesicles.. Curr Opin Cell Biol.

[39] Wilhelm C, Browaeys J, Ponton A, Bacri JC (2003). Rotational magnetic particles microrheology: the Maxwellian case.. Phys Rev E.

[40] Watanabe TM, Higuchi H (2007). Stepwise Movements in Vesicle Transport of HER2 by Motor Proteins in Living Cells.. Biophys J.

[41] Caspi A, Granek R, Elbaum M (2002). Diffusion and directed motion in cellular transport.. Phys Rev E.

